# The Impact of Urbanization on Avian Communities During the Breeding Season in the Huanghuai Plain of China

**DOI:** 10.1002/ece3.71255

**Published:** 2025-04-07

**Authors:** Meiting Liu, Jiayi Shi, Ziruo Zhang, Xinyi Zhang, Xiaohan Li, Ruohui Tang, Chunna Zhang, Siyu Wu, Chenfang Wu, Junpo Zhu, Zhirong He, Yujia Sun, Yuehuan Wang, Supen Wang, Na Zhao

**Affiliations:** ^1^ Collaborative Innovation Center of Recovery and Reconstruction of Degraded Ecosystem in Wanjiang Basin Co‐Founded by Anhui Province and Ministry of Education College of Ecology and Environment, Anhui Normal University Wuhu China; ^2^ The Anhui Provincial Key Laboratory of Biodiversity Conservation and Ecological Security in the Yangtze River Basin College of Life Science, Anhui Normal University Wuhu China; ^3^ College of Life Sciences, Nanjing Agricultural University Nanjing China

**Keywords:** bird species diversity, breeding season, functional characteristics, Huanghuai plain, urbanization

## Abstract

The noise pollution, habitat loss, and human disturbance caused by urbanization have damaged bird communities. Research on the relationship between urbanization and birds has predominantly focused on highly urbanized areas, with relatively few studies in underdeveloped urbanized areas. Here, we conducted bird surveys along the urban–rural continuum by utilizing 150 line transects within a 51,385 km^2^ area from June to August in 2022 and 2023, aiming to explore the impact of urbanization on bird species diversity and functional traits during the breeding season in the Huanghuai Plain of China. We found significant differences in species diversity and functional traits among three habitats along the urban–rural continuum (i.e., urban, suburban, and rural). Additionally, a measure combining several aspects of urbanization (the urban synthetic index) had significant negative correlations with species richness and the Shannon‐Wiener index, while it had no significant correlation with functional traits. We then assessed that the environmental noise, the distance to the county center, and the proportion of building area within a 250‐m radius were critical factors affecting species diversity, as well as environmental noise and the distance to the county center were the best predictors for functional traits. The composition and proportions of diets and nest types of birds were similar across the urban, suburban, and rural habitats. Our study highlights the importance of environmental noise, the distance to the county center, and the building index in protecting urban birds in the Huanghuai Plain. The research findings filled a gap in the study area regarding the relationship between urbanization and avian communities based on the urban–rural continuum.

## Graphical abstract

Our study reported the effect of urbanization on the bird communities during the breeding season and underscored the significance of these urbanization‐related factors in the protection of birds within the study area. This finding addressed the gap in research that had been previously identified in the Huanghuai Plain.

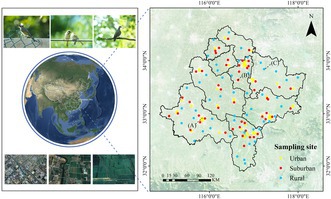

## 1 Introduction

Urbanization drastically transforms natural landscapes, leading to a decline in biodiversity (Marzluff et al. 2012; McDonald et al. 2020). By 2030, urban land is projected to expand to 1.2 million km^2^ (Seto et al. 2012), with approximately 5.2 billion urban residents living in urban areas around the world (United Nations 2018). Urbanization is concurrent with the reduction in biodiversity (Shochat et al. 2010; Lizée et al. 2011; Aronson et al. 2014; Sol et al. 2017; Evans et al. 2018; Palacio et al. 2018; Barbosa et al. 2020; Santos, Wiederhecker et al. 2024). Numerous studies have found that there is a decline in animal diversity with the increase of impermeable surface from rural to urban regions (McKinney 2002; Blair 2004; Evans et al. 2018; Piano et al. 2020; Hastedt and Tietze 2023; Vaz et al. 2023; Santos, Wiederhecker et al. 2024). For example, the species‐area relationship indicates that the expansion of urban land (e. g., artificial grass, paved surfaces, and buildings) results in the loss of plant species richness (Blair and Launer 1997; McKinney 2008). The plant structure in cities often tends to be simplified (Marzluff and Ewing 2008), which negatively affects animal diversity, as their diversity concerns the complexity and species richness of vegetation (Savard et al. 2000). Moreover, urbanization is a major driver of biotic homogenization (McKinney 2006). Despite being a minority view, certain research findings argue that moderate levels of urbanization can support peak species diversity, thus supporting the Intermediate Disturbance Hypothesis (IDH) (Lepczyk et al. 2008; Callaghan, Bino et al. 2019; Callaghan, Major et al. 2019; Leveau 2021). Support for the IDH in medium urbanized areasindicates that areas with moderate urbanization disturbance can sustain the highest bird diversity, while emphasizing the importance, such as parks and suburban areas in maintaining bird diversity. This insight would guide conservation efforts to protect these critical refuges and inform urban design, promoting a balance between bird diversity and human development. Urban areas can exhibit high spatial habitat heterogeneity, which may provide niches for certain species (Savard et al. 2000). Additionally, the rate of invasion and establishment of exotic species exceeds the rate of loss, which can temporarily increase local species richness, but it often leads to biotic homogenization and ecosystem instability (McKinney 2002, 2006). In conclusion, urban environments change the landscape and, as a result, affect biodiversity.

Birds are widely distributed in urban ecosystems. Their ecological characteristics (e.g., active behavior and variable vocalizations) make them more easily observable compared to other biological taxa. Given their sensitivity to environmental changes, the population density and diversity of birds serve as critical indicators of environmental quality and biodiversity in urban ecosystems (Gregory et al. 2003). Consequently, birds are often chosen as focal species in urbanization studies (Rodrigues et al. 2018; Leveau and Leveau 2020; Neate‐Clegg et al. 2023; Duan et al. 2024; Santos, Wiederhecker et al. 2024; Zhong et al. 2024). While this topic has been explored in various regions worldwide, research in China remains limited, with most studies mainly focused on developed areas (Chen, Ding et al. 2000; Chen, Zhang et al. 2022; Chen, Liu et al. 2023; Chen, Zhang et al. 2023; Zhang and Huang 2018; Leveau and Leveau 2020; Batisteli et al. 2021, 2025; Leveau 2021; Sun et al. 2022; Santos et al. 2023, 2025; Duan et al. 2024).

Studies have shown that urbanization exerts multidimensional impacts on birds. Firstly, urban birds adapt to novel selective pressures in urban environments through morphological changes, such as body size and beak length (Evans, Gaston et al. 2009; Evans, Newson et al. 2009; Santos, et al. 2023; Santos, Pompermaier et al. 2024). Secondly, urban birds typically experience higher physiological stress, which makes them more prone to physiological issues and significantly increases the prevalence of diseases (Partecke et al. 2006; Deviche et al. 2023; Santos, Pompermaier et al. 2024). Furthermore, urbanization profoundly influences the diets, reproductive behaviors, and migratory patterns of birds (Shochat et al. 2006; Tryjanowski et al. 2016; Chen, Liu et al. 2023; Chen, Zhang et al. 2023). It is a primary driver of both the decline in avian species richness and the biotic homogenization in bird communities (McKinney 2006; Sol et al. 2014; Leveau et al. 2015). Additionally, the distance to the city center is a key factor influencing avian diversity and distribution. Areas closer to the city center typically exhibit lower bird diversity due to higher levels of habitat fragmentation and human activity, while suburban and rural areas often support richer avian diversity (Marzluff et al. 2001; McKinney 2002). Specifically, the increased building density, heightened environmental noise, and elevated human disturbance caused by urbanization have significant impacts on the habitats and lifestyles of birds. The increase in impervious surface area (i.e., buildings, roads, and other built‐up structures) leads to habitat loss, which is a primary driver of biodiversity decline, as artificial structures remove and degrade critical habitats for many animal species (Seto et al. 2012; Leveau and Leveau 2020). The increase in building density in urban areas affects avian diversity (Evans, Gaston et al. 2009; Evans, Newson et al. 2009; Souza et al. 2019; Wang and Zhou 2022; Yu et al. 2025), foraging behavior (Lowry et al. 2013; Sol et al. 2014; Santos et al. 2025), and reproductive behavior (Batisteli et al. 2021; Chen et al. 2024; Corsini and Szulkin 2025). It may even increase the likelihood of bird mortality due to collisions with buildings (Loss et al. 2014; Hager et al. 2017; Elmore et al. 2021). Environmental noise, as one of the primary characteristics of urbanization, negatively impacts birds, including diversity (Cai 2012; Proppe et al. 2013; Morelli et al. 2021), vocal communication (Patricelli and Blickley 2006; Hawkins et al. 2020; Toki et al. 2021), foraging and migration behaviors (Kunz et al. 2007; Tryjanowski et al. 2013; Francis 2015; Sweet et al. 2022), and reproduction (Injaian et al. 2019; Senzaki et al. 2020; Chen, Liu et al. 2023; Chen, Zhang et al. 2023) of birds in urban. Such birds are particularly vulnerable to traffic noise, with a notable decline in population density in areas with high traffic volumes (Cai 2012); Birds also experience a decline in clutch size with increasing noise levels (Senzaki et al. 2020). Meanwhile, the growth of urban residents has led to an intensification of human disturbance (e.g., environmental noise and habitat destruction), resulting in significant negative impacts on birds (Bradley and Altizer 2007; Seto et al. 2012; Van Doren et al. 2017; Senzaki et al. 2020; Corsini and Szulkin 2025). However, urban areas also provide green spaces that birds treat as “refuge habitats” such as urban parks and artificial lakes (Sandström et al. 2006; Leveau and Leveau 2016; Callaghan, Bino et al. 2019; Callaghan, Major et al. 2019). Therefore, further research is needed to enhance our understanding of the multifaceted impact of urbanization on avian communities.

As mentioned above, the various impacts of urbanization on birds imply that urbanization introduces novel selective pressures for avian species, which may lead to changes in their life story traits (Ibáñez‐Álamo and Soler 2010; Lowry et al. 2013; Mascarenhas et al. 2023). Life history refers to the sequence of stages that an organism goes through from birth to death, including all the events and processes that occur during its lifetime, which can effectively reflect the adaptive plasticity of species (Martin 2004). Urban habitats filter out ecologically specialized species, retaining those with specific traits or combinations of traits that are adapted to urban environments (Zhong et al. 2024). Generally, urban‐adapted birds are identified as ecological generalists, characterized by a set of functional traits that enhance their survival in urbanization areas. For instance, urban birds tend to have a broader diet, enabling them to utilize anthropogenic food resources such as garbage, bird feed, and fruits from ornamental plants, which enhances their ability to survive in urban environments (Bonier et al. 2007; Callaghan, Bino et al. 2019; Callaghan, Major et al. 2019; Palacio 2020). Nesting site selection is also significantly influenced by urbanization (Lan et al. 2021; Chen et al. 2024; Batisteli et al. 2025). Urban birds tend to build nests on buildings, bridges, or trees, avoiding nesting on the ground to reduce predation risk (Evans, Gaston et al. 2009; Evans, Newson et al. 2009; Conole and Kirkpatrick 2011; Dale et al. 2015). Urban birds exhibit larger clutch sizes, allowing them to adapt to the instability of urban habitats (Callaghan, Bino et al. 2019; Callaghan, Major et al. 2019; Senzaki et al. 2020). The distribution breadth is another key trait, as urban‐adapted birds typically have broader distributions and stronger dispersal abilities (Møller 2009; Zhong et al. 2024). Although it is generally believed that urban birds tend to have smaller body sizes, potentially due to enhanced metabolic efficiency and environmental adaptability or limited resource availability in urban areas (Liker et al. 2008; Meillère et al. 2015; Merckx et al. 2018; Cooper et al. 2022). Despite extensive evidence on the ecological and life history characteristics of birds related to urbanization, the results are often contradictory. For example, some studies have found that urban birds with medium size are more likely to adapt to urban environments, but no clear trend of body size changes has been observed (Conole and Kirkpatrick 2011; Møller et al. 2015; Seress and Liker 2015), while other studies suggest that urban birds may increase in body size as the abundance of resources in urban areas (Liker and Bókony 2009; Evans et al. 2010; Seress and Liker 2015; Alberti et al. 2017). Therefore, it is essential to investigate which functional traits are most closely related to urban adaptation, which helps us understand the relationship between urban birds and urbanization.

The Huanghuai Plain, the most populous plain in China, features flat terrain, numerous rivers, and a temperate climate. It has developed into a significant political, economic, cultural, and transportation hub as it has urbanized. However, prior research on the effects of urbanization on birds has primarily focused on the largest developed areas (e.g., Shanghai, Beijing, and Hangzhou), with a notable lack of studies in regions with lower urbanization in the Huanghuai Plain of China. Therefore, this experiment will fill a gap in the effect of urbanization on birds in the Huanghuai Plain. This study will provide insights into the differences in adaptive traits of birds across various levels of urbanization, offering recommendations for biodiversity conservation.

Given the greater stability of the bird communities during the breeding season, this period was chosen for the study. Here, this study explored the relationship between birds and urbanization by examining species diversity and functional traits during the breeding season within the Huanghuai Plain of China. We hypothesized that the urbanization gradient along the rural–urban continuum has a profound impact on birds in the study area. To test this hypothesis, we established three predictions regarding the relationship between urbanization and bird communities: (1) if urbanization affects species diversity, then species diversity will exhibit a significant difference among three habitats (i.e., urban, suburban, and rural), with the lowest in the urban habitat. (2) if birds adapt to urban environments, then urban birds will have smaller body mass, larger clutch size, and wider distribution breadth than other habitats. Additionally, urban birds are expected to be arboreal and have diverse diets. (3) we expect that species diversity and functional traits (i.e., body size and distribution breadth) will have significant negative correlations with the urbanization synthetic index, including the building index, environmental noise, and the disturbance index, while will have significant positive correlations with the distance to the county center. However, clutch size will exhibit the opposite pattern compared to the variables mentioned above.

## 2 Methods

### 2.1 Study Area

**FIGURE 1:**
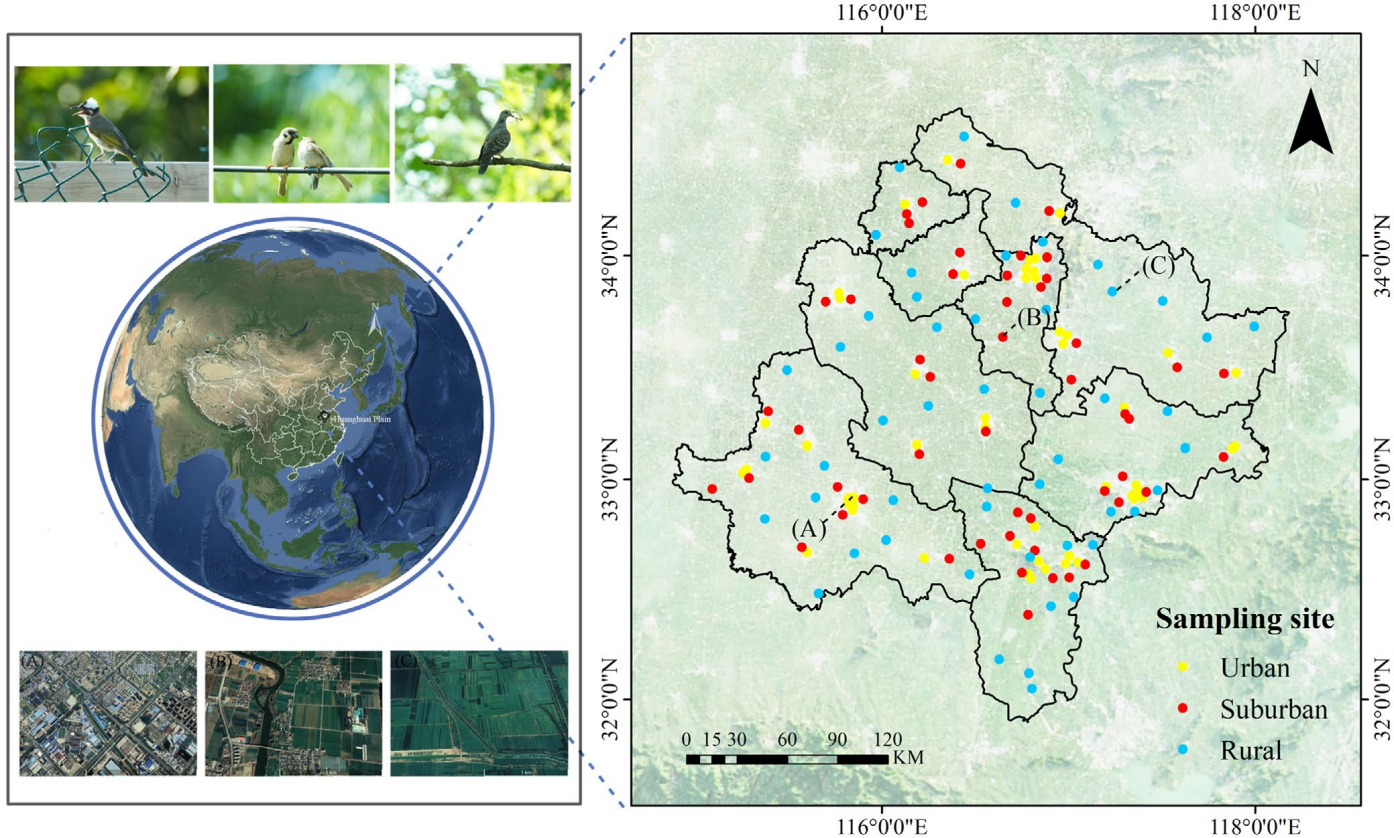
The map of study area and the sampling sites of field surveys in the Huanghuai Plain. The dots indicate the location of line transects among three habitats (i.e., urban, suburban, and rural). On the below are three photos from Google Earth to demonstrate the urbanization gradient along the rural–urban continuum in this study. (A) Urban, (B) suburban, (C) rural.

We conducted this study in the southern of Huanghuai Plain in China (33°16′ ~ 34°14 N, 116°23′ ~ 117°02′ E), situated in the northern of the Anhui province and certain regions within Henan province. The terrain of the study area is predominantly characterized by plains and scattered low‐lying hills and mountains, with altitudes generally below 200 m. The climate belongs to the warm temperate semi‐moist monsoon climate, which the climate is mild and rainy, with an average annual temperature ranging from 14°C to 16°C and annual precipitation of between 800 and 1000 mm. The vegetation type primarily consists of warm temperate deciduous broadleaf forests, complemented by coniferous forests and shrublands, providing a relatively diverse habitat composition (Figure 1).

### 2.2 Line Transects and Habitats Classification

We defined three habitats (i.e., urban, suburban, and rural) based on the classification system that researchers have previously established about the global rural–urban continuum in 2020 (1‐km resolution) (Li et al. 2023). Specifically, the vector layer representing the boundary of the study area was used to clip it in ArcGIS 10.8, getting the vector layer of the rural–urban continuum classification system for the study area at first. The vector layer includes nine classifications (i.e., urban, town, village, cropland, grassland, woodland, wild, water, and ice/snow) (Li et al. 2023). Then, we excluded the vector layer of water and classified the remaining eight vector layers. Finally, the urban layer was categorized as urban habitat, the layers of town and village belonging to suburban habitat, and the remaining layers belonging to rural habitat. Urbanization varies across different regions, resulting in a gradient of urbanization ranging from urban to suburban and rural, with the urbanization decreasing as the distance from the urban area increases in the study area. According to the mapping of the rural–urban continuum of the study area, we have pre‐established 150 fixed 1‐km line transects based on the principles of randomness and accessibility and took into account the terrain, vegetation, hydrology, and land use of the habitats to ensure representative sampling for each habitat. Finally, we redistributed some line transects that were originally located in the water area or distributed in areas overlapping with the water layer to each habitat and ensured that there were 50 transects in each habitat.

### 2.3 Field Surveys

Bird surveys were conducted by two experienced researchers during 4 h from dawn and 3 h before sunset in good weather (e.g., no wind and rain) in summer from June to August 2022. The observers walked along each line transect at a constant speed of approximately 1.0 km/h—2.0 km/h and used binoculars for direct observation to identify birds. Additionally, observers also utilized a camera (SONY Alpha 7III with a 200–600 mm telephoto lens) to document birds that were unidentifiable within a 50‐m radius while not included those flying over the head. From June to August 2023, we carried out repeat bird surveys by using the same methods. The composition of land use types of each line transect did not change during our surveys.

### 2.4 Data Collection

#### 2.4.1 Avian Species Identification and Classification

The identification and classification of birds were based on *A checklist on the Classification and Distribution of Birds of China* (Zheng 2023) and *A Field Guide to the Birds of China* (Mackinnon et al. 2000). The levels of endangerment and conservation status are based on *The List of National Key Protected Wildlife*, *The IUCN Red List of Threatened Species* (IUCN; https://www.iucnredlist.org/) and *The Red List of Biodiversity in China: Vertebrates*.

#### 2.4.2 Functional Characteristics Collection

To investigate the impact of urbanization on the functional traits of birds in the Huanghuai Plain, we selected 5 ecological and life history characteristics (i.e., body mass, diet, clutch size, nest site, and distributed provinces) for our study (Wang, Song et al. 2021; Wang, Zhu et al. 2021).

#### 2.4.3 Urbanization Characteristics Variables

Compared to earlier studies on urbanization, researchers used a single variable or indicator to substitute the level of urbanization (Marzluff et al. 2001). It appears to be insufficient in fully representing the true level of urbanization within the study area. Some studies have employed an urbanization synthetic index to conduct related research to mitigate negative effects (Chen et al. 2022; Wang and Zhou 2022). Therefore, we also selected an urbanization synthetic index that includes four main factors (i.e., the building index, the environment noise, the disturbance index, and the distance to the city center) (Chen et al. 2000; Wang et al. 2008).

We imported all line transects into ArcGIS 10.8, and established four buffer zones around each line transect, with a radius of 250 m, 500 m, 1000 m, and 2000 m, respectively, to estimate the proportion of buildings surrounding the lines (Bolger et al. 1997). The Building Index (BI) was calculated by summing the weighted building proportions within each buffer zone. The weights were assigned based on the assumption that the influence of buildings decreases with increasing distance from the line transects. Specifically, the building index is calculated as follows: The Building Index (BI) = 250 m of building area% × 1 + 500 m of building area% × 0.5 + 1000 m of building area% × 0.25 + 2000 m of building area% × 0.125 (Wang et al. 2009).

While conducting field surveys along each line transect, we measured the environmental noise using a decibel meter. Measurements were conducted once during each of the three time periods: morning, midday, and evening, with each measurement lasting for 10 min. The average of the noise values obtained from each line transect was then taken as the environmental noise (Wang and Zhou 2022). Meanwhile, we also carried out the collection of the disturbance index. The observation at each line transect, and human traffic was recorded, with each observation lasting for 10 min. The average value was then adopted. The study divided human disturbance into five levels, with Level 1 indicating the absence of humans; Level 2 indicating human traffic of 1–2 people per minute; Level 3 indicating human traffic of 3–7 people per minute; Level 4 indicating human traffic of 8–17 people per minute; and Level 5 indicating human traffic of 18 people per minute or more (Chen et al. 2000).

Our study divided the study area into county‐level units, with the government hall at each county serving as the central point. The distance to the county center (DCC) was measured as a straight line from line transects to the government hall of each county (km) with a Google map.

Following other studies, an urbanization synthetic index (USI) for our study is as follows: USI = BI × 100/2 + EN + DI × 20 + 100/DCC (Chen et al. 2000; Wang et al. 2008; Wang and Zhou 2022). We adjusted parameters value to the range of 0–100, with higher numbers representing a higher level of urbanization (Chen et al. 2000; Wang et al. 2008). In detail, the BI value ranged from 0 to 2; we standardized it by multiplying by 100 and then dividing by 2. The EN value ranged from 0 to 100, so it remained unchanged. The DI value ranged from 1 to 5, so it was multiplied by 20; the DCC value from 0 to 60, so it was taken the reciprocal and multiplied by 100.

### 2.5 Data Analyses

**FIGURE 2:**
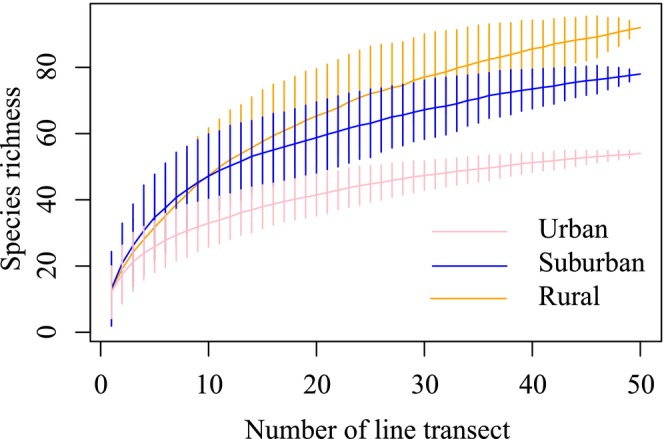
Species accumulation curves of bird surveys in study area.

We conducted a preliminary analysis by drawing species accumulation curves to evaluate the sampling effort. The result indicated that our field survey was sufficiently comprehensive, allowing for subsequent analyses (Figure 2).

For each line transect, we calculated the species richness, the abundance of every species, the Shannon‐Wiener diversity index (Shannon and Weaver 1998), the Pielou evenness index (Pielou 1966), and the Simpson diversity index (Simpson 1949). Prior to analyses, the Pielou evenness index and species richness were transformed by taking the square root to achieve normality, while the USI was log‐transformed for the same purpose. Then a one‐way analysis of variance (ANOVA) was employed to test for differences among three habitats, followed by Tukey's Honest Significant Difference (HSD) test for pairwise comparisons. The Pielou evenness index did not exhibit a significant difference among the three habitats, the rest of the diversity indices showed significant differences (Table S1). Therefore, we then conducted linear mixed models (LMMs) to explore the relationship between the urbanization synthetic index and bird species diversity, with the USI as the fixed effect and the research sites as the random factors. In addition, we carried out the Pearson correlation analysis to avoid interference in the results due to multicollinearity among the various urbanization factors and species diversity. To avoid interference in the results due to multicollinearity among the various urbanization factors and species diversity, we conducted Pearson correlation analysis. While the correlation between the building index and environmental noise was relatively high (*r* = 0.745), both indices exhibited significant relationships with the diversity index. Therefore, we retained them in subsequent analyses to ensure a comprehensive assessment of urbanization impacts (Table S3). This experiment also employed linear mixed models to determine which factors in the USI (i.e., BI, EN, DI, DCC) influenced species diversity and their relative importance, with the study sites serving as the random factor. We employed the Akaike Information Criterion (AIC) to determine which of the building index proportions within four different radiuses had the greatest impact, and we integrated the lowest AIC value into linear mixed models for further analyses (Table S2). The analysis yielded a series of models. We then selected and ranked models based on the cumulative difference values of the Akaike Information Criterion (ΔAICc < 2). The candidate models are considered to have substantial support (Burnham and Anderson 2002). Next, we calculated the Akaike weight (*w*_*i*_) for each candidate model based on the ΔAICc values. The Akaike weight (*w*_*i*_) refers to the probability that the model is the best model in candidate models (*w*_*i*_ > 0.9) (Burnham and Anderson 2002; Guthery et al. 2003). However, the Akaike weights indicated that none of the candidate models were the best model (Table S5). Subsequently, model averaging was conducted to obtain the relative importance (*w*_*i*_) of each parameter within a 95% confidence interval, along with the model estimates and standard errors (SE), to mitigate uncertainty in model selection. We employed the same procedures and methods to investigate the relationship between urbanization and functional traits (i.e., body mass, clutch size, and distribution breadth) of birds (Tables S4, S6). Diet and nest site are categorical data. We first calculated the number of species across three habitats based on distinct diets and nest sites, and then employed a chi‐square test to analyze the differences in these variables among the three habitats. All analyses were performed in R 4.3.3 (R Core Team 2024), and the significance level was set at *α* = 0.05.

## 3 Results

### 3.1 Bird Community Composition

This survey recorded a total of 106 bird species during two breeding seasons, which belong to 14 orders and 45 families including 60 passerine species and 46 nonpasserine species. There were 53 species in urban habitats, 77 species in suburban habitats, and 91 species in rural habitats. Among the recorded species, there were 5 listed as Near Threatened (NT) on the *The Red List of Biodiversity in China: Vertebrates* (i.e., *Charadrius placidus* , *Circus cyaneus* , *Accipiter trivirgatus* , *Falco peregrinus* , *Terpsiphone incei*) and 6 were classified as Class II Key Protected Wildlife in China (i.e., *Platalea leucorodia* , *Accipiter virgatus, Circus cyaneus, Accipiter nisus, Accipiter trivirgatus, Falco peregrinus*) (Table S7).

### 3.2 Variation in Species Diversity With Urbanization

**FIGURE 3:**
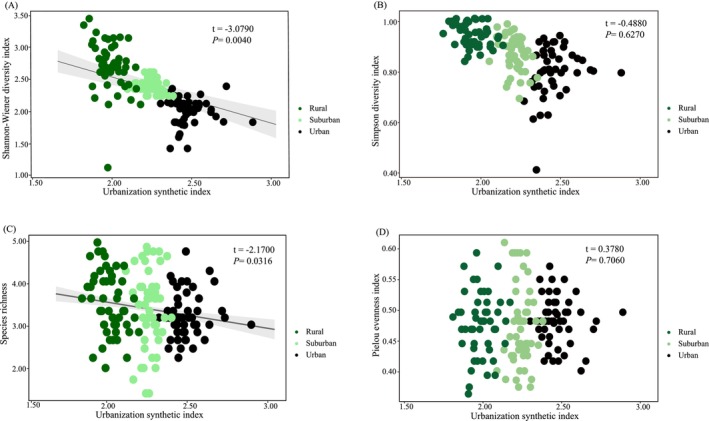
The relationship between species diversity and urbanization synthetic index (USI), log transformed: (A) Shannon‐Wiener diversity; (B) Simpson diversity index; (C) Species richness, square root transformed; (D) Pielou evenness index, square root transformed.

The results of linear mixed models showed that the urbanization synthetic index exhibited significant negative correlations with the Shannon‐Wiener diversity index (*p* = 0.0342) and the species richness(*p* = 0.0316), while it showed no significant relationship with the Simpson diversity index (*p* = 0.6270) and the Pielou evenness index (*p* = 0.7060) (Figure 3; Table 1). The urbanization synthetic index has no significant relationship with the species richness of near‐threatened and nationally key protected birds (*p* = 0.3420) (Table 1).

**TABLE 1:** Results of linear mixed models (LMMs) of species diversity of birds with urbanization synthetic index. *p* < 0.05 is marked in bold.

Diversity index	Intercept	Estimate ± SE	*t*	*p*
Shannon‐Wiener diversity index	3.8473	−0.6969 ± 0.2264	−3.0790	**0.0040**
Simpson diversity index	0.9356	−0.0322 ± 0.0661	−0.4880	0.6270
Species richness	4.6526	−0.5872 ± 0.2706	−2.1700	**0.0316**
Pielou evenness index	0.4602	0.0073 ± 0.0193	0.3780	0.7060
Species richness of near threatened and nationally key protected birds	0.7327	−0.2763 ± 0.2469	−1.1190	0.3420

### 3.3 The Models Assessing the Impacts of Urbanization on Avian Species Diversity

The model average results from the linear mixed model analyses indicated key factors influencing the Shannon‐Wiener diversity index of birds include the proportion of building area within a 250‐m radius (*w*_*i*_ = 1.0000, Estimate = −0.4827 ± 0.1568SE, *Z* = 3.7550, *p* = 0.0002), the environmental noise (*w*_*i*_ = 0.8000, Estimate = −0.0061 ± 0.0030SE, *Z* = 2.0190, *p* = 0.0435), and the distance to the county center (*w*_*i*_ = 1.0000, Estimate = 0.0074 ± 0.0027SE, *Z* = 2.7100, *p* = 0.0067). A key factor influencing species richness is environmental noise (*w*_*i*_ = 1.0000, Estimate = −0.0145 ± 0.0060SE, *p* = 0.0150). The Simpson index and the Pielou evenness index showed no significant relationships with the urbanization factors. With the increase in the distance to the county center, the species richness of near threatened and nationally key protected birds significantly increases (*w*_*i*_ = 0.7700, Estimate = 0.0087 ± 0.0044SE, *p* = 0.0484) (Table 2).

**TABLE 2:** Results of linear mixed models (LMMs) equivalent model average of urbanization factors on species diversity of birds (ΔAICc < 2), *p* < 0.05 is marked in bold.

Diversity index	Urbanization factors	Weight (*W*_*i*_)	Estimate	SE	*Z*	*p*
Shannon‐Wiener diversity index	Proportion of building area—250 m	1.0000	−0.4827	0.1568	3.7550	**0.0002**
	Environmental noise	0.8000	−0.0061	0.0030	2.0190	**0.0435**
	Disturbance index	0.5000	−0.0423	0.0325	1.3010	0.1934
	The distance to county center	1.0000	0.0074	0.0027	2.7100	**0.0067**
Simpson diversity index	Environmental noise	0.7700	−0.0007	0.0004	1.6570	0.0975
	Disturbance index	0.3800	−0.0060	0.0052	1.1490	0.2504
	The distance to county center	0.1400	−0.0003	0.0005	0.5590	0.5763
	Proportion of building area—2000 m	0.1300	0.0072	0.0194	0.3740	0.7086
Species richness	Environmental noise	1.0000	−0.0145	0.0060	2.4310	**0.0150**
	The distance to county center	0.3200	−0.0073	0.0067	1.0910	0.2750
	Disturbance index	0.1900	−0.0403	0.0800	0.5050	0.6140
Pielou evenness index	Disturbance index	0.2250	0.0081	0.0077	1.0560	0.2910
	Environmental noise	0.2240	0.0006	0.0006	1.0540	02920
	Proportion of building area—1000 m	0.1920	0.0237	0.0263	0.8990	0.3690
Species richness of near threatened and nationally key protected birds	Proportion of building area—2000 m	0.5900	−0.3324	0.1906	1.7310	0.0834
	The distance to county center	0.7700	0.0087	0.0044	1.9740	**0.0484**
	Disturbance index	0.8900	0.0957	0.0505	1.8810	0.0600
	Environmental noise	0.3800	−0.0058	0.0048	1.1940	0.2324

### 3.4 The Models Assessing the Impacts of Urbanization on Functional Traits

**FIGURE 4:**
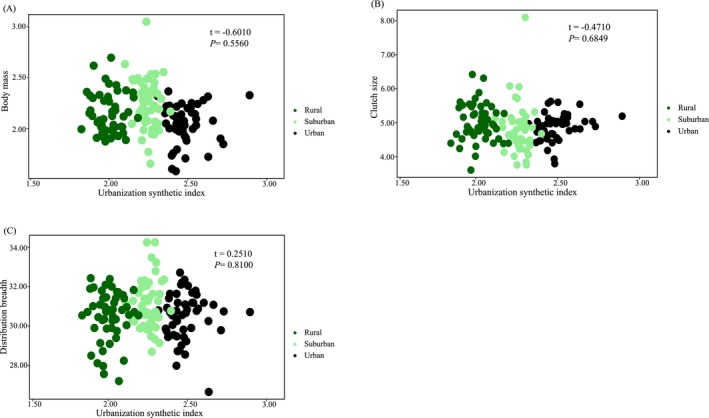
The relationship between species diversity and urbanization synthetic index (USI), log transformed: (A) Body mass, log transformed; (B) Clutch size; (C) Distribution breadth.

**FIGURE 5:**
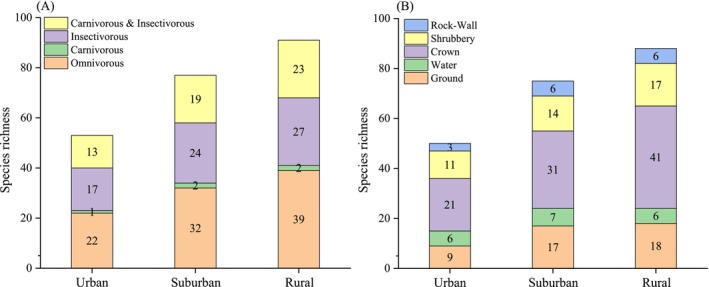
Comparison of traits of bird among the three habitats (i.e., urban, suburban, and rural) including diet and nest site (A, B). The numbers in the figures represent species richness.

The body mass, clutch size, and distribution breadth have shown no significant correlations with the urbanization synthetic index (Figure 4; Table S8). However, according to the results of the model average, there was a significant negative correlation between clutch size and the proportion of building area within a 2000‐m radius (*w*_*i*_ = 0.8700, Estimate = −0.3118 ± 0.1538SE, *p* = 0.0444). The distribution breadth showed a significant negative correlation with the distance to the county center (*w*_*i*_ = 0.8500, Estimate = −0.0249 ± 0.0108SE, *p* = 0.0223). In terms of body mass, there was no significant correlation with urbanization factors (Table S9). From the perspective of the dietary groups to which bird species belong, the composition of the diet was similar across the urban, suburban, and rural: the order of bird species by diet, from most to least abundant, is: omnivorous, insectivorous, carnivorous and insectivorous, and carnivorous. The number of bird species with each nest site was as follows: crown, ground, shrubbery, water, and rock wall (Figure 5). The results of the chi‐square test revealed that only the species richness of omnivorous birds showed significant differences across the three habitats among four diets (*χ* ^2^ = 6.122, df = 2, *p* = 0.047). Regarding nest sites, the species richness of birds nesting in shrubbery (*χ* ^2^ = 8.219, df = 2, *p* = 0.016) and on rock wall (*χ* ^2^ = 7.721, df = 2, *p* = 0.021) exhibited significant differences among the three habitats (Table S10).

## 4 Discussion

Our findings revealed that a total of 106 bird species were recorded during the breeding season. The species richness, abundance, and Shannon‐Wiener diversity index were the highest in rural habitat, followed by suburban habitat, and the lowest in urban habitat, showing a decrease along the urbanization gradient. This pattern is not only the same as our first prediction but also consistent with other outcomes of previous studies (McKinney 2002, 2008; Chace and Walsh 2006; Shochat et al. 2010; Batáry et al. 2018; Chamberlain et al. 2017; Mao et al. 2019; Piano et al. 2020; Hastedt and Tietze 2023; Vaz et al. 2023). This aligns with the results of the linear mixed model analyses, which showed that species richness and the Shannon‐Wiener index had a significant negative relationship with the urbanization synthetic index, whereas other diversity indices showed no significant correlation with the urbanization synthetic index. This could be attributed to the increase in species richness and new species as urbanization decreases, while the dominant species remain similar among three habitats, with a few species maintaining their dominance in communities (McCarthy, 2004) Moreover, in contrast to the other two habitats, suburban habitats were classified as moderate disturbance. Nevertheless, species richness in suburban habitats was lower than in rural areas, which contradicts the intermediate disturbance hypothesis. This discrepancy contrasts with the findings of previous research (Wang and Zhou 2022; Duan et al. 2024) and suggests that the disturbance level has exceeded the threshold for promoting diversity. Additionally, the lack of habitat heterogeneity in suburban areas could limit the establishment of diverse species communities, leading to lower species richness compared to rural habitats. This difference may be attributed to the combined effects of various factors such as regional biodiversity, the extent of urbanization, and management policies, which may lead to diverse outcomes (Wang et al. 2012).

Li et al. (2020) simulated the distribution habitats of 1111 bird species across China, revealing that 220 species tended to select rural as their habitats. It is consistent with our findings that the average value of species richness, as well as the Shannon‐Wiener and the Simpson diversity index, was highest in three habitats. This is closely related to the higher openness and abundant food resources in rural habitats, especially during the period of crop maturity and harvest (Crampton et al. 2011; Rosin et al. 2016). The insects, weed seeds, and leftover grains in the grasslands within the agricultural fields provided rich food for birds. Consequently, rural habitat is characterized by a prevalence of omnivorous and insectivorous bird species (Figure 5). Additionally, the rural landscapes had many paddy fields, rivers, and artificial irrigation channels within the study area, which have attracted many birds primarily feeding on carnivorous diets, with a lot of aquatic birds. This finding is consistent with previous research on the effects of artificial landscapes on breeding bird communities (Moreno‐Mateos et al. 2009; López‐Pomares et al. 2015; Giosa et al. 2018; Deguchi et al. 2020; Wang, Song et al. 2021; Wang, Zhu et al. 2021). However, the Pielou evenness index of the three habitats was low, due to the dominance of human‐associated species (e.g., *Hirundo rustica* , *Cecropis daurica* , *Passer montanus* , *Spilopelia chinensis* ) within the avian communities, which account for the majority of the total bird individuals (i.e., urban [67.35%], suburban [42.48%], and rural [54.36%]).

There was a significant difference in the average body mass of birds among the three habitats, with urban birds having the smallest (Table S1). The most common raptors (Accipitridae) in urban centers are smaller (Cooper et al. 2022). The clutch size in urban was at an intermediate level, and a higher number of clutches can enhance the population growth potential. This is inconsistent with some studies that have found that areas with higher urbanization have larger clutch sizes (Callaghan, Bino et al. 2019; Callaghan, Major et al. 2019; Senzaki et al. 2020). This may be influenced by additional factors such as resource availability, predation pressure, or breeding strategies, which could moderate the overall clutch size in urban environments. Species with high reproductive capabilities could rebuild their populations more rapidly following disturbance (Larsen et al. 2005). Among the 53 bird species recorded in urban habitats, the omnivorous birds are the most numerous (Figure 5). It could be evergreen plants, fruiting trees in urban parks, and residential waste generated by the community serving as food sources for omnivorous birds (Marzluff and Ewing 2001). The artificial lakes within the park, along with the planting of aquatic vegetation, provide habitats for large waterfowl and various waders, offering them spaces for survival and reproduction (Zhang et al. 2020). Moreover, the selection of nesting sites is crucial for the reproduction and survival of birds. Urban birds predominantly consist of arboreal species, whereas ground‐nesting birds, particularly those that construct nests on the ground or in shrubbery, tend to favor locations distant from urban environments (Dale et al. 2015). In this study, nearly half of the urban birds utilized crown sites, consistent with previous research findings. Complex plant communities enhance the safety of nesting sites for urban birds and ensure an abundant food supply, which are key factors determining whether birds can inhabit urban areas (Strohbach et al. 2013; Beninde et al. 2015; Threlfall et al. 2016, 2017; Aronson et al. 2017). Therefore, exploring the relationship between the life story of birds and urbanization helps us have useful implications for the protection of birds in urban ecosystems (Martin and Roper 1988). It also implies that certain life story traits may enhance their ability to colonize urban environments. For instance, arboreal nesting provides greater protection from ground predators and human disturbance, while a larger clutch size enhances population resilience to urban environments pressures. Similarly, species with wide distribution ranges and generalist diets, particularly omnivores, may better exploit the diverse and unpredictable food resources in cities. By highlighting these traits as potential urban adaptations, our findings also contribute to a broader understanding of how urbanization shapes avian community composition beyond the Huanghuai Plain.

Urban birds face various survival pressures, and the relationship between urbanization and birds is currently of great concern to researchers (Isaksson 2018; Møller et al. 2015; Leveau et al. 2021; Chen, Liu et al. 2023; Chen, Zhang et al. 2023). Our study employed an urbanization synthetic index and constructed linear mixed models aiming to investigate which specific urbanization factors affect bird diversity. The results were consistent with our third prediction. They exhibited a significant negative correlation between environmental noise and the Shannon‐Wiener index and species richness. Some research has demonstrated that environmental noise has adverse effects on bird fitness, survival, and reproduction (Habib et al. 2007; Gross et al. 2010; Schroeder et al. 2012). Anthropogenic noise reduces bird species richness and diversity in urban parks (Perillo et al. 2017). In addition, a negative relationship between maximum point‐count noise and avian species richness was found in research that untangled the role of anthropogenic noise on bird species richness in a Neotropical city (Carral‐Murrieta et al. 2020). These are consistent with our findings. The distance to the county center is often used as one of the indicators to measure the level of urbanization, with longer distances suggesting lower urbanization (Chen et al. 2000; Wang et al. 2004). As the distance to the county center increases, there is a decline in urbanization and human disturbance, concurrently leading to a higher proportion of natural habitats, which contributes to an enhancement of bird diversity within the distant area (Chen et al. 2000; Wang et al. 2004). Our findings yielded similar outcomes that distance to the county center exhibited a significant positive correlation with species richness and diversity. For example, the species richness of nationally key protected birds and near threatened is also higher in rural habitat than other habitats, indicating that these species prefer habitats with less disturbance in the Huanghuai Plain. In addition, As the distance to the county center decreased, the distribution breadth became broader. It indicated that birds in urban habitats exhibited a wider distribution range and were better adapted to habitat heterogeneity because urban habitats may also provide the resources and space they require. Previous findings have shown that the increase in the proportion of buildings within small‐scale wetlands has resulted in the loss of natural habitats, thereby posing a challenge to the survival of certain bird species (Wang and Zhou 2022). In this study, the Shannon‐Wiener index and clutch size had a significant negative correlation with the proportion of building area within a 250‐m radius. Yet, it also indicated that an increase in building proportion may have harmful effects on birds. In conclusion, our study underscores the critical role of urbanization factors (i.e., BI, EN, DI, and DCC) in shaping bird communities within urban areas of the study area, emphasizing the need for targeted conservation strategies to mitigate adverse effects and promote the coexistence of urban birds with human development.

Based on our findings, we propose the following conservation strategies to mitigate the negative impacts of urbanization on bird communities: (1) preserving and restoring natural habitats, such as wetlands and grasslands, within urban and suburban areas; (2) enhancing urban green spaces and nesting opportunities; (3) mitigating noise pollution and habitat fragmentation; (4) promoting biodiversity‐friendly agricultural and suburban landscapes; (5) promoting public awareness and community involvement in bird conservation efforts.

## 5 Conclusions

Through field surveys of birds, this study has preliminarily compiled a checklist of breeding bird species in the Huanghuai Plain. Species diversity (i.e., Shannon‐wiener index, and species richness) has a negative relationship with USI by using LMMs. The results also showed that environmental noise, the distance to the county center, and the proportion of building area within a 250‐m radius were key factors affecting bird species diversity. Environmental noise and the distance to the county center were critical factors affecting functional traits. Urban birds tended to have smaller body mass, larger clutch sizes, diverse diets, and arboreal nesting sites in our study. Therefore, we understood that these functional traits are associated with urban‐adapted birds, as well as highlight the importance of environmental noise, the distance to the county center, and the building index for the protection of urban birds in Huanghuai Plain. The research findings filled the gap in the study area regarding the relationship between urbanization and the avian community. However, there is a slight deficiency in fully understanding the distribution pattern of bird diversity in the Huanghuai Plain, and efforts should be strengthened in the future to investigate the bird resources.

## Author Contributions

**Meiting Liu:** data curation (equal), formal analysis (equal), writing – original draft (equal). **Jiayi Shi:** data curation (equal), formal analysis (equal). **Ziruo Zhang:** data curation (equal). **Xinyi Zhang:** data curation (equal). **Xiaohan Li:** data curation (equal). **Ruohui Tang:** data curation (equal). **Chunna Zhang:** data curation (equal). **Siyu Wu:** data curation (equal). **Chenfang Wu:** data curation (equal). **Junpo Zhu:** data curation (equal). **Zhirong He:** data curation (equal). **Yujia Sun:** data curation (equal). **Yuehuan Wang:** data curation (equal). **Supen Wang:** funding acquisition (equal), writing – review and editing (equal). **Na Zhao:** funding acquisition (equal), writing – review and editing (equal).

## Conflicts of Interest

The authors declare no conflicts of interest.

## Supporting information


Tables S1–S10.

## Data Availability

All data and R code used in the study are available in Dryad. https://doi.org/10.5061/dryad.0p2ngf2b6.
