# Supplementary material for: The Impact of Urbanization on Avian Communities During the Breeding Season in the Huanghuai Plain of China
Source: Ecol Evol. 2025 Apr 7;15(4):e71255. doi: 10.1002/ece3.71255 (PMC11975628; doi:10.1002/ece3.71255)
Supplement: Supplementary file 1 — Tables S1–S10. [file ECE3-15-e71255-s001.docx]

**Appendix Table S1.** Differences in bird species diversity and functional traits across the three habitats (i.e., urban, suburban, and rural).

| Variables | Urban  (Mean±SE) | Suburban  (Mean±SE) | Rural  (Mean±SE) | Tukey HSD | Anova |
| --- | --- | --- | --- | --- | --- |
| Shannon-Wiener diversity index | 1.965±0.217 | 2.353±0.099 |  | *P*<0.001 | F _(2,147)_ =87.982  *P* < 0.001 |
|  | 1.965±0.217 |  | 2.656±0.390 | *P*<0.001 |  |
|  |  | 2.353±0.099 | 2.656±0.390 | *P*<0.001 |  |
|  |  |  |  |  |  |
| Simpson diversity index | 0.847±0.100 | 0.871±0.099 |  | *P =* 0.398 | F _(2,147)_ =2.740  *P* = 0.068 |
|  | 0.847±0.100 |  | 0.889±0.051 | *P =* 0.054 |  |
|  |  | 0.871±0.099 | 0.889±0.051 | *P =* 0.555 |  |
|  |  |  |  |  |  |
| Pielou evenness index | 0.232±0.037 | 0.227±0.062 |  | *P =* 0.566 | F _(2,147)_ =0.524  *P* = 0.594 |
|  | 0.232±0.037 |  | 0.229±0.047 | *P =* 0.829 |  |
|  |  | 0.227±0.062 | 0.229±0.047 | *P =* 0.901 |  |
|  |  |  |  |  |  |
| Species richness | 10.840±3.835 | 11.940±5.658 |  | *P =* 0.559 | F _(2,147)_ =7.493  *P* = 0.001 |
|  | 10.840±3.835 |  | 12.780±5.060 | *P =* 0.001 |  |
|  |  | 11.940±5.658 | 12.780±5.060 | *P =* 0.020 |  |
|  |  |  |  |  |  |
| Body mass | 132.754±51.081 | 204.713±148.391 |  | *P* =0.001 | F _(2,147)_ =8.830  *P* < 0.001 |
|  | 132.754±51.081 |  | 136.241±57.546 | *P* =0.990 |  |
|  |  | 204.713±148.391 | 136.241±57.546 | *P* =0.001 |  |
|  |  |  |  |  |  |
| Clutch size | 4.837±0.373 | 4.765±0.692 |  | *P* =0.795 | F _(2,147)_ =1.703  *P* =0.186 |
|  | 4.837±0.373 |  | 4.966±0.541 | *P* =0.471 |  |
|  |  | 4.765±0.692 | 4.966±0.541 | *P* =0.167 |  |
|  |  |  |  |  |  |
| Distribution breadth | 30.325±0.373 | 30.919±1.202 |  | *P* =0.039 | F _(2,147)_ =4.093  *P* =0.019 |
|  | 30.325±0.373 |  | 30.318±1.250 | *P* =1.000 |  |
|  |  | 30.919±1.202 | 30.318±1.250 | *P* =0.037 |  |

**Appendix Table S2.** Comparison of the model support for the impact of building index on species diversity and functional traits at 4 different scales. The lowest AIC values were marked in bold.

|  | 250m of building area% (AIC) | 500m of building area% (AIC) | 1000m of building area % (AIC) | 2000m of building area % (AIC) |
| --- | --- | --- | --- | --- |
| Shannon-Wiener  diversity index | **36.690** | 39.193 | 38.444 | 40.967 |
| Simpson diversity index | -461.410 | -461.420 | -461.810 | **-461.820** |
| Pielou evenness index | -276.960 | -277.020 | **-277.670** | -277.110 |
| Species richness | 335.240 | 335.130 | **334.300** | 335.550 |
| Body mass | **-51.606** | -52.231 | -49.248 | -48.512 |
| Clutch size | 251.370 | 252.060 | 251.540 | **251.250** |
| Distribution breadth | 492.890 | 492.680 | **492.660** | 492.770 |
| Species richness of near threatened and Class II Key Protected birds | 199.49 | 198.69 | 198.99 | **197.91** |

**Appendix Table S3. The result of Pearson correlation analysis among species diversity and urbanization factors.**

|  | Building index | Environmental noise | Disturbance  index | The distance to county center | Shannon-Wiener index | Simpson index | Pielou evenness index | Species richness |
| --- | --- | --- | --- | --- | --- | --- | --- | --- |
| Building index | 1.000 |  |  |  |  |  |  |  |
| Environmental noise | r=0.745  *p*<0.001 | 1.000 |  |  |  |  |  |  |
| Disturbance  index | r=0.607  *p*<0.001 | r=0.681  *p*<0.001 | 1.000 |  |  |  |  |  |
| The distance to county center | r=-0.651  *p*<0.001 | r=-0.639  *p*<0.001 | r=-0.517  *p*<0.001 | 1.000 |  |  |  |  |
| Shannon-Wiener index | r=-0.684  *p*<0.001 | r=-0.624  *p*<0.001 | r=-0.529  *p*<0.001 | r=0.588  *p*<0.001 | 1.000 |  |  |  |
| Simpson index | r=-0.085  *p*=0.299 | r=-0.166  *p*=0.042 | r=-0.150  *p*=0.068 | r=0.071  *p*<0.001 | r=0.117  *p*=0.154 | 1.000 |  |  |
| Pielou evenness index | r=0.038  *p*=0.645 | r=0.087  *p*=0.290 | r=-0.087  *p*=0.289 | r=-0.016  *p*=0.845 | r=-0.156  *p*=0.056 | r=-0.504  *p*<0.001 | 1.000 |  |
| Species richness | r=-0.277  *p*=0.001 | r=-0.345  *p*<0.001 | r=-0.331  *p*<0.001 | r=0.185  *p*=0.023 | r=0.259  *p*=0.001 | r=0.409  *p*<0.001 | r=-0.767  *p*<0.001 | 1.000 |

**Appendix Table S4. The result of Pearson correlation analysis among functional traits and urbanization factors.**

|  | Building index | Environmental noise | Disturbance  index | The distance to county center | Body mass | Clutch size | Distribution breadth |
| --- | --- | --- | --- | --- | --- | --- | --- |
| Building index | 1.000 |  |  |  |  |  |  |
| Environmental noise | r=0.745  *p*<0.001 | 1.000 |  |  |  |  |  |
| Disturbance  index | r=0.607  *p*<0.001 | r=0.681  *p*<0.001 | 1.000 |  |  |  |  |
| The distance to county center | r=0.651  *p*<0.001 | r=0.639  *p*<0.001 | r=-0.517  *p*<0.001 | 1.000 |  |  |  |
| Body mass | r= -0.102  *p*=0.213 | r= -0.004  *p*=0.963 | r= -0.049  *p*<0.001 | r= -0.051  *p*=0.533 | 1.000 |  |  |
| Clutch size | r=0.175  *p*=0.032 | r= -0.069  *p*=0.400 | r=0.007  *p*=0.931 | r=0.134  *p*=0.101 | R= -0.146  *P*=0.075 | 1.000 |  |
| Distribution breadth | r=-0.004  *p*=0.963 | r=0.007  *p*=0.935 | r=0.073  *p*=0.372 | r= -0.132  *p*=0.107 | r=0.467  *p*<0.001 | r=0.027  *p*=0.745 | 1.000 |

**Appendix Table S5.** All models of urbanization factors on species diversity of bird based on LMM (best models ΔAICc ＜2 are shown in bold).

| Diversity index | Model | df | *logLik* | AICc | ΔAICc | *W*_i_ |  |
| --- | --- | --- | --- | --- | --- | --- | --- |
| Shannon-Wiener diversity index | BI+EN+DCC | 5 | -17.152 | 44.700 | **0.000** | 0.424 |  |
|  | BI+EN+DI+DCC | 6 | -16.542 | 45.700 | **0.950** | 0.264 |  |
|  | BI+DI+DCC | 5 | -18.078 | 46.600 | **1.850** | 0.168 |  |
|  | BI+DCC | 5 | -19.855 | 48.000 | 3.270 | 0.083 |  |
|  | BI+EN | 4 | -20.839 | 50.000 | 5.230 | 0.031 |  |
|  | BI+EN+DI | 5 | -20.016 | 50.400 | 5.730 | 0.024 |  |
|  | BI+DI | 4 | -22.984 | 54.200 | 9.520 | 0.004 |  |
|  | EN+DI+DCC | 5 | -22.825 | 56.100 | 11.350 | 0.001 |  |
|  | EN+DCC | 4 | -24.396 | 57.100 | 12.350 | 0.001 |  |
|  | BI | 3 | -25.988 | 58.100 | 13.420 | 0.001 |  |
|  | DI+DCC | 4 | -29.121 | 66.500 | 21.800 | 0.000 |  |
|  | EN+DI | 4 | -29.703 | 67.700 | 22.960 | 0.000 |  |
|  | EN | 3 | -32.210 | 70.600 | 25.860 | 0.000 |  |
|  | DCC | 3 | -37.468 | 81.100 | 36.380 | 0.000 |  |
|  | DI | 3 | -44.655 | 95.500 | 50.750 | 0.000 |  |
|  | NULL | 2 | -69.253 | 142.600 | 97.870 | 0.000 |  |
|  |  |  |  |  |  |  |  |
| Simpson diversity index | EN | 3 | 236.404 | -466.600 | **0.000** | 0.208 |  |
|  | DI | 3 | 236.001 | -465.800 | **0.810** | 0.139 |  |
|  | EN+DI | 4 | 236.595 | -464.900 | **1.730** | 0.088 |  |
|  | EN+DCC | 4 | 236.566 | -464.900 | **1.790** | 0.085 |  |
|  | BI+EN | 4 | 236.477 | -464.700 | **1.970** | 0.078 |  |
|  | BI+DI | 4 | 236.012 | -436.700 | 2.900 | 0.049 |  |
|  | DI+DCC | 4 | 236.005 | -436.700 | 2.910 | 0.049 |  |
|  | BI | 3 | 234.910 | -463.700 | 2.990 | 0.047 |  |
|  | EN+DI+DCC | 5 | 236.818 | -463.200 | 3.420 | 0.038 |  |
|  | DCC | 3 | 234.682 | -463.200 | 3.450 | 0.037 |  |
|  | BI+EN+DI | 5 | 236.703 | -463.000 | 3.650 | 0.034 |  |
|  | BI+EN+DCC | 5 | 236.590 | -462.800 | 3.880 | 0.030 |  |
|  | BI+DI+DCC | 5 | 236.029 | -461.600 | 5.000 | 0.017 |  |
|  | BI+DCC | 4 | 234.946 | -461.6000 | 5.030 | 0.017 |  |
|  | BI+EN+DI+DCC | 6 | 236.861 | -461.100 | 5.510 | 0.013 |  |
|  |  |  |  |  |  |  |  |
| Species richness | EN | 3 | -161.107 | 328.400 | **0.000** | 0.258 |  |
|  | EN+DCC | 4 | -160.493 | 329.300 | **0.880** | 0.166 |  |
|  | EN+DI | 4 | -160.976 | 330.200 | **1.850** | 0.102 |  |
|  | BI+EN | 4 | -161.093 | 330.500 | 2.080 | 0.091 |  |
|  | EN+DI+DCC | 5 | -160.257 | 330.900 | 2.550 | 0.072 |  |
|  | BI+EN+DCC | 5 | -160.365 | 331.100 | 2.770 | 0.065 |  |
|  | DI | 3 | -162.631 | 331.400 | 3.050 | 0.056 |  |
|  | BI+EN+DI | 5 | -160.972 | 332.400 | 3.980 | 0.035 |  |
|  | BI | 3 | -163.150 | 332.500 | 4.080 | 0.033 |  |
|  | BI+DI | 4 | -162.148 | 332.600 | 4.190 | 0.032 |  |
|  | BI+EN+DI+DCC | 6 | -160.172 | 332.900 | 4.550 | 0.027 |  |
|  | DI+DCC | 4 | -162.605 | 333.500 | 5.110 | 0.020 |  |
|  | BI+DI+DCC | 5 | -161.886 | 334.200 | 5.810 | 0.014 |  |
|  | BI+DCC | 4 | -163.104 | 334.500 | 6.100 | 0.012 |  |
|  | DCC | 3 | -164.883 | 335.900 | 7.550 | 0.006 |  |
|  |  |  |  |  |  |  |  |
| Pielou evenness index | NULL | 2 | 142.418 | -280.800 | **0.000** | 0.201 |  |
|  | DI | 3 | 142.990 | -279.800 | **0.940** | 0.126 |  |
|  | EN | 3 | 142.988 | -279.800 | **0.940** | 0.125 |  |
|  | BI | 3 | 142.833 | -279.500 | **1.250** | 0.107 |  |
|  | DCC | 3 | 142.438 | -278.700 | 2.040 | 0.072 |  |
|  | EN+DCC | 4 | 143.187 | -278.100 | 2.660 | 0.053 |  |
|  | EN+DI | 4 | 143.098 | -277.900 | 2.840 | 0.049 |  |
|  | DI+DCC | 4 | 143.077 | -277.900 | 2.880 | 0.048 |  |
|  | BI+DI | 4 | 143.061 | -277.800 | 2.910 | 0.047 |  |
|  | BI+EN | 4 | 143.018 | -277.800 | 2.990 | 0.045 |  |
|  | BI+DCC | 4 | 142.926 | -277.600 | 3.180 | 0.041 |  |
| 续表 |  |  |  |  |  |  |  |
| Diversity index | | Model | df | *logLik* | AICc | ΔAICc | *W*_i_ |
|  | EN+DI+DCC | 5 | 143.348 | -276.300 | 4.480 | 0.021 |  |
|  | BI+EN+DCC | 5 | 143.284 | -276.200 | 4.600 | 0.020 |  |
|  | BI+DI+DCC | 5 | 143.268 | -276.100 | 4.640 | 0.020 |  |
|  | BI+EN+DI | 5 | 143.112 | -275.800 | 4.950 | 0.017 |  |
|  | BI+EN+DI+DCC | 6 | 143.415 | -274.200 | 6.510 | 0.008 |  |
|  |  |  |  |  |  |  |  |
| Species richness of near threatened and Class II Key Protected birds | BI+DI+DCC | 5 | -92.211 | 194.800 | 0.000 | 0.174 |  |
|  | EN+DI+DCC | 5 | -92.650 | 195.700 | 0.880 | 0.112 |  |
|  | DI+DCC | 4 | -93.731 | 195.700 | 0.900 | 0.111 |  |
|  | BI+DI | 4 | -93.904 | 196.100 | 1.250 | 0.093 |  |
|  | BI+EN+DI+DCC | 6 | -91.814 | 196.200 | 1.380 | 0.087 |  |
|  | DCC | 3 | -95.060 | 196.300 | 1.450 | 0.084 |  |
|  | BI+EN+DI | 5 | -92.994 | 196.400 | 1.570 | 0.079 |  |
|  | BI | 3 | -95.345 | 196.900 | 2.020 | 0.063 |  |
|  | BI+ DCC | 4 | -94.478 | 197.200 | 2.390 | 0.053 |  |
|  | EN+DI | 4 | -94.807 | 197.900 | 3.050 | 0.038 |  |
|  | EN+DCC | 4 | -94.993 | 198.300 | 3.420 | 0.031 |  |
|  | BI+EN | 4 | -95.296 | 198.900 | 4.030 | 0.023 |  |
|  | EN | 3 | -96.532 | 199.200 | 4.390 | 0.019 |  |
|  | BI+EN+DCC | 5 | -94.464 | 199.300 | 4.510 | 0.018 |  |
|  | NULL | 2 | -98.351 | 200.800 | 5.950 | 0.009 |  |
|  | DI | 3 | -98.350 | 202.900 | 8.030 | 0.003 |  |

**Appendix Table S6.** All models of urbanization factors on functional traits of bird based on LMM (best models ΔAICc ＜ 2 are shown in bold).

| Functional traits | Model | df | *logLik* | AICc | ΔAICc | *W*_i_ |
| --- | --- | --- | --- | --- | --- | --- |
| Body mass | BI+DCC | 4 | 26.070 | -43.900 | **0.000** | 0.135 |
|  | NULL | 2 | 23.916 | -43.700 | **0.110** | 0.127 |
|  | BI+DI+DCC | 5 | 26.988 | -43.600 | **0.300** | 0.116 |
|  | BI+DI | 4 | 25.890 | -43.500 | **0.360** | 0.112 |
|  | BI | 3 | 24.516 | -42.900 | **1.000** | 0.082 |
|  | BI+EN+DCC | 5 | 26.301 | -42.200 | **1.680** | 0.058 |
|  | BI+EN | 4 | 25.210 | -42.100 | **1.720** | 0.057 |
|  | DCC | 3 | 24.113 | -42.100 | **1.800** | 0.055 |
|  | DI | 3 | 24.098 | -42.000 | **1.830** | 0.054 |
|  | EN | 3 | 23.917 | -41.700 | 2.190 | 0.045 |
|  | BI+EN+DI | 5 | 26.041 | -41.700 | 2.200 | 0.045 |
|  | BI+EN+DI+DCC | 6 | 27.005 | -41.400 | 2.440 | 0.040 |
|  | EN+DI | 4 | 24.293 | -40.300 | 3.550 | 0.023 |
|  | EN+DCC | 4 | 24.282 | -40.300 | 3.570 | 0.023 |
|  | DI+DCC | 4 | 24.166 | -40.100 | 3.810 | 0.020 |
|  | EN+DI+DCC | 5 | 24.565 | -38.700 | 5.150 | 0.010 |
| Clutch size | BI | 3 | -121.643 | 249.500 | **0.000** | 0.169 |
|  | BI+DI | 4 | -120.685 | 249.600 | **0.200** | 0.153 |
|  | BI+DI+DCC | 5 | -120.159 | 250.700 | **1.280** | 0.089 |
|  | DCC | 3 | -122.330 | 250.800 | **1.370** | 0085 |
|  | BI+EN | 4 | -121.393 | 251.100 | **1.610** | 0.075 |
|  | BI+DCC | 4 | -121.484 | 251.200 | **1.790** | 0.069 |
|  | NULL | 2 | -123.698 | 251.500 | 2.030 | 0.061 |
|  | DI+DCC | 4 | -121.714 | 251.700 | 2.250 | 0.055 |
|  | BI+EN+DI | 5 | -120.685 | 251.800 | 2.340 | 0.053 |
|  | BI+EN+DCC | 5 | -120.968 | 252.400 | 2.900 | 0.040 |
|  | BI+EN+DI+DCC | 6 | -120.110 | 252.800 | 3.360 | 0.032 |
|  | EN | 3 | -123.338 | 252.800 | 3.390 | 0.031 |
|  | EN+DCC | 4 | -122.294 | 252.900 | 3.410 | 0.031 |
|  | DI | 3 | -123.694 | 253.600 | 4.100 | 0.022 |
|  | EN+DI+DCC | 5 | -121.641 | 253.700 | 4.250 | 0.020 |
|  | EN+DI | 4 | -122.923 | 254.100 | 4.670 | 0.016 |
| Distribution breadth | BI+DCC | 4 | -240.639 | 489.600 | **0.000** | 0.185 |
|  | DCC | 3 | -242.066 | 490.300 | **0.740** | 0.128 |
|  | EN+DCC | 4 | -241.287 | 490.800 | **1.290** | 0.097 |
|  | NULL | 2 | -243.384 | 490.800 | **1.300** | 0.097 |
|  | BI+DI+DCC | 5 | -240.356 | 491.100 | **1.570** | 0.084 |
|  | BI+EN+DCC | 5 | -240.525 | 491.500 | **1.910** | 0.071 |
|  | DI | 3 | -242.978 | 492.100 | 2.570 | 0.051 |
|  | EN+DI+DCC | 5 | -240.903 | 492.200 | 2.670 | 0.049 |
|  | DI+DCC | 4 | -242.064 | 492.400 | 2.850 | 0.045 |
|  | BI+EN+DI+DCC | 6 | -239.956 | 492.500 | 2.950 | 0.042 |
|  | BI | 3 | -243.314 | 492.800 | 3.240 | 0.037 |
|  | EN | 3 | -243.381 | 492.900 | 3.370 | 0.034 |
|  | BI+DI | 4 | -242.410 | 493.100 | 3.540 | 0.032 |
|  | EN+DI | 4 | -242.714 | 493.700 | 4.150 | 0.023 |
|  | BI+EN | 4 | -243.205 | 494.700 | 5.130 | 0.014 |
|  | BI+EN+DI | 5 | -242.385 | 495.200 | 5.630 | 0.011 |

**Appendix Table S7.** The checklist of the breeding birds in the study area.

| Species | Habitat | National  Key Protection  Level | IUCN  Species  Threatened  level | Red List of Biodiversity in China | Endemic  Species  Of China |
| --- | --- | --- | --- | --- | --- |
| **Galliformes** |  |  |  |  |  |
| 1、Phasianidae |  |  |  |  |  |
| *Phasianus colchicus* | a, b, c |  | LC | LC |  |
| *Bambusicola thoracicus* | a |  | LC | LC | 🗸 |
| **Anseriformes** |  |  |  |  |  |
| 1、Anatidae |  |  |  |  |  |
| *Anas zonorhyncha* | a, b, c |  | LC | LC |  |
| *Anas platyrhynchos* | b, c |  | LC | LC |  |
| **Podicipediformes** |  |  |  |  |  |
| 1、Podicedidae |  |  |  |  |  |
| *Tachybaptus ruficollis* | a, b, c |  | LC | LC |  |
| *Podiceps cristatus* | b |  | LC | LC |  |
| **Columbiformes** |  |  |  |  |  |
| 1、Columbidae |  |  |  |  |  |
| *Streptopelia orientalis* | a, b, c |  | LC | LC |  |
| *Spilopelia chinensis* | a, b, c |  | LC | LC |  |
| **Cuculiformes** |  |  |  |  |  |
| 1、Cuculidae |  |  |  |  |  |
| *Eudynamys scolopaceus* | a, c |  | LC | LC |  |
| *Cuculus canorus* | a, b, c |  | LC | LC |  |
| *Cuculus micropterus* | b, c |  | LC | LC |  |
| **Gruiformes** |  |  |  |  |  |
| 1、Rallidae |  |  |  |  |  |
| *Gallinula chloropus* | a, b, c |  | LC | LC |  |
| *Fulica atra* | a, b, c |  | LC | LC |  |
| *Zapornia akool* | a, b |  | LC | LC |  |
| **Charadriiformes** |  |  |  |  |  |
| 1、Charadriidae |  |  |  |  |  |
| *Charadrius dubius* | b |  | LC | LC |  |
| *Vanellus cinereus* | b, c |  | LC | LC |  |
| *Charadrius placidus* | b, c |  | LC | NT |  |
| 2、Recurvirostridae |  |  |  |  |  |
| *Himantopus himantopus* | b, c |  | LC | LC |  |
| 3、Scolopacidae |  |  |  |  |  |
| *Tringa nebularia* | b |  | LC | LC |  |
| *Actitis hypoleucos* | a |  | LC | LC |  |
| *Tringa glareola* | c |  | LC | LC |  |
| 4、Scolopacidae |  |  |  |  |  |
| *Gallinago gallinago* | b, c |  | LC | LC |  |
| *Tringa erythropus* | b |  | LC | LC |  |
| 5、Laridae |  |  |  |  |  |
| *Chlidonias hybrida* | a, b, c |  | LC | LC |  |
| *Sterna hirundo* | a, b, c |  | LC | LC |  |
| **Pelecaniformes** |  |  |  |  |  |
| 1、Threskiornithidae |  |  |  |  |  |

| 续表 |  |  |  |  |  |
| --- | --- | --- | --- | --- | --- |
| Species | Habitat | National  Key Protection  Level | IUCN  Species  Threatened  level | Red List of Biodiversity in China | Endemic  Species  Of China |
| *Platalea leucorodia* | b | II | LC | LC |  |
| 2、Ardeidae |  |  |  |  |  |
| *Egretta garzetta* | a, b, c |  | LC | LC |  |
| *Ardea intermedia* | a, b, c |  | LC | LC |  |
| *Ardea alba* | b, c |  | LC | LC |  |
| *Nycticorax nycticorax* | a, b, c |  | LC | LC |  |
| *Ardea cinerea* | a, b, c |  | LC | LC |  |
| *Butorides striata* | a, c |  | LC | LC |  |
| *Ardeola bacchus* | a, b, c |  | LC | LC |  |
| *Bubulcus coromandus* | a, b, c |  | LC | LC |  |
| *Ixobrychus sinensis* | a, b, c |  | LC | LC |  |
| **Accipitriformes** |  |  |  |  |  |
| 1、Accipitridae |  |  |  |  |  |
| *Accipiter virgatus* | b, c | II | LC | LC |  |
| *Circus cyaneus* | c | II | LC | NT |  |
| *Accipiter nisus* | c | II | LC | LC |  |
| *Accipiter trivirgatus* | c | II | LC | NT |  |
| **Bucerotiformes** |  |  |  |  |  |
| 1、Upupidae |  |  |  |  |  |
| *Upupa epops* | a, b, c |  | LC | LC |  |
| **Coraciiformes** |  |  |  |  |  |
| 1、Alcedinidae |  |  |  |  |  |
| *Alcedo atthis* | a, b, c |  | LC | LC |  |
| *Ceryle rudis* | b, c |  | LC | LC |  |
| **Piciformes** |  |  |  |  |  |
| 1、Picidae |  |  |  |  |  |
| *Yungipicus canicapillus* | a, b, c |  | LC | LC |  |
| *Dendrocopos major* | b |  | LC | LC |  |
| *Picus canus* | c |  | LC | LC |  |
| **Falconiformes** |  |  |  |  |  |
| 1、Falconidae |  |  |  |  |  |
| *Falco peregrinus* | b, c | II | LC | NT |  |
| **Passeriformes** |  |  |  |  |  |
| 1、Campephagidae |  |  |  |  |  |
| *Pericrocotus cantonensis* | c |  | LC | LC |  |
| 2、Laniidae |  |  |  |  |  |
| *Lanius cristatus* | a, b, c |  | LC | LC |  |
| *Lanius schach* | a, b, c |  | LC | LC |  |
| *Lanius bucephalus* | c |  | LC | LC |  |
| *Lanius tigrinus* | b, c |  | LC | LC |  |
| 3、Corvidae |  |  |  |  |  |
| *Cyanopica cyanus* | a, b, c |  | LC | LC |  |
| *Pica serica* | a, b, c |  | LC | LC |  |
| *Corvus corone* | b |  | LC | LC |  |
| 4、Paridae |  |  |  |  |  |
| *Parus major* | a, b, c |  | LC | LC |  |
| 5、Cisticolidae |  |  |  |  |  |
| *Prinia inornata* | b |  | LC | LC |  |
| 6、Acrocephalidae |  |  |  |  |  |
| *Acrocephalus orientalis* | a, b |  | LC | LC |  |
| 7、Phylloscopidae |  |  |  |  |  |
| *Phylloscopus borealis* | c |  | LC | LC |  |
| 8、Pycnonotidae |  |  |  |  |  |
| *Spizixos semitorques* | a, b, c |  | LC | LC |  |
| *Pycnonotus sinensis* | a, b, c |  | LC | LC |  |
| *Pycnonotus xanthorrhous* | c |  | LC | LC |  |
| 9、Hirundinidae |  |  |  |  |  |
| *Hirundo rustica* | a, b, c |  | LC | LC |  |
| *Cecropis daurica* | a, b, c |  | LC | LC |  |
| 续表 |  |  |  |  |  |
| Species | Habitat | National  Key Protection  Level | IUCN  Species  Threatened  level | Red List of Biodiversity in China | Endemic  Species  Of China |
| 10、Cettiidae |  |  |  |  |  |
| *Horornis fortipes* | c |  | LC | LC |  |
| *Horornis canturians* | a, b, c |  | LC | LC |  |
| 11、Aegithalidae |  |  |  |  |  |
| *Aegithalos glaucogularis* | a, b, c |  | LC | LC | 🗸 |
| *Aegithalos concinnus* | b, c |  | LC | LC |  |
| 12、Paradoxornithidae |  |  |  |  |  |
| *Sinosuthora webbiana* | a, b, c |  | LC | LC |  |
| 13、Zosteropidae |  |  |  |  |  |
| *Zosterops simplex* | b, c |  | LC | LC |  |
| 14、Oriolidae |  |  |  |  |  |
| *Oriolus chinensis* | a, b, c |  | LC | LC |  |
| 15、Dicruridae |  |  |  |  |  |
| *Dicrurus macrocercus* | a, b, c |  | LC | LC |  |
| *Dicrurus hottentottus* | c |  | LC | LC |  |
| 16、Leiothrichidae |  |  |  |  |  |
| *Pterorhinus perspicillatus* | a, b |  | LC | LC |  |
| 17、Cinclidae |  |  |  |  |  |
| *Cinclus pallasii* | b |  | LC | LC |  |
| 18、Sturnidae |  |  |  |  |  |
| *Acridotheres cristatellus* | a, b, c |  | LC | LC |  |
| *Spodiopsar sericeus* | a, b, c |  | LC | LC |  |
| *Spodiopsar cineraceus* | a, b, c |  | LC | LC |  |
| 19、Turdidae |  |  |  |  |  |
| *Turdus hortulorum* | b, c |  | LC | LC |  |
| *Turdus mandarinus* | a, b, c |  | LC | LC |  |
| *Turdus cardis* | b, c |  | LC | LC |  |
| *Myophonus caeruleus* | a, c |  | LC | LC |  |
| 20、Muscicapidae |  |  |  |  |  |
| *Copsychus saularis* | a, b, c |  | LC | LC |  |
| *Phoenicurus auroreus* | a, b, c |  | LC | LC |  |
| *Ficedula zanthopygia* | c |  | LC | LC |  |
| 21、Monarchidae |  |  |  |  |  |
| *Terpsiphone incei* | b, c |  | LC | NT |  |
| 22、Estrildidae |  |  |  |  |  |
| *Lonchura striata* | c |  | LC | LC |  |
| 23、Passeridae |  |  |  |  |  |
| *Passer montanus* | a, b, c |  | LC | LC |  |
| 24、Motacillidae |  |  |  |  |  |
| *Motacilla alba* | a, b, c |  | LC | LC |  |
| *Motacilla tschutschensis* | c |  | LC | LC |  |
| *Dendronanthus indicus* | c |  | LC | LC |  |
| *Anthus cervinus* | c |  | LC | LC |  |
| *Anthus rubescens* | c |  | LC | LC |  |
| *Anthus rufulus* | c |  | LC | LC |  |
| 25、Fringillidae |  |  |  |  |  |
| *Fringilla montifringilla* | b, c |  | LC | LC |  |
| *Eophona migratoria* | a, b, c |  | LC | LC |  |
| *Eophona personata* | a, c |  | LC | LC |  |
| *Chloris sinica* | a, b, c |  | LC | LC |  |
| *Spinus spinus* | c |  | LC | LC |  |
| *Coccothraustes coccothraustes* | c |  | LC | LC |  |
| 26、Emberizidae |  |  |  |  |  |
| *Emberiza pusilla* | b, c |  | LC | LC |  |
| *Emberiza spodocephala* | b, c |  | LC | LC |  |
| *Emberiza cioides* | b, c |  | LC | LC |  |
| *Emberiza rutila* | c |  | LC | LC |  |
| 续表 |  |  |  |  |  |
| Species | Habitat | National  Key Protection  Level | IUCN  Species  Threatened  level | Red List of Biodiversity in China | Endemic  Species  Of China |
| *Emberiza elegans* | c |  | LC | LC |  |
| *Emberiza chrysophrys* | c |  | LC | LC |  |
| 27、Alaudidae |  |  |  |  |  |
| *Alauda gulgula* | b |  | LC | LC |  |
| Habitat: a: Urban; b: Suburban; c: Rural. National Key Protection Level: National second-class key protected species. LC: Least Concern; NT: Near Threatened. | | | | | |

Table S8 Results of linear mixed models (LMMs) of functional traits of birds with urbanization synthetic index. *P* ＜ 0.05 is marked in bold.

| Functional traits | Intercept | Estimate ± SE | t | *P* |
| --- | --- | --- | --- | --- |
| Body mass | 2.3554 | -0.1001±0.1665 | -0.6010 | 0.5560 |
| Clutch size | 5.1314 | -0.1256±0.2669 | -0.4710 | 0.6849 |
| Distribution breadth | 30.0698 | 2.2057±0.8200 | -0.2510 | 0.8100 |

Table S9 Results of linear mixed models (LMMs) equivalent model average of urbanization factors on functional traits of bird (ΔAICc ＜ 2), P ＜ 0.05 is marked in bold.

| Functional traits | Urbanization factors | Weight (*W_i_*) | Estimate | SE | Z | *P* |
| --- | --- | --- | --- | --- | --- | --- |
| Body mass | Proportion of building area – 250m | 0.7000 | -0.1481 | 0.0894 | 1.6570 | 0.0975 |
|  | The distance to county center | 0.4600 | -0.0028 | 0.0021 | 1.3410 | 0.1800 |
|  | Disturbance index | 0.3500 | 0.0279 | 0.0278 | 1.2250 | 0.2204 |
|  | Environmental noise | 0.1400 | 0.0019 | 0.0021 | 0.8820 | 0.3779 |
| Clutch size | Proportion of building area – 2000m | 0.8700 | -0.3118 | 0.1538 | 1.8400 | **0.0444** |
|  | The distance to county center | 0.3800 | 0.0049 | 0.0049 | 1.0010 | 0.3618 |
|  | Disturbance index | 0.3800 | 0.0780 | 0.0543 | 1.4370 | 0.1508 |
|  | Environmental noise | 0.1200 | 0.0034 | 0.0049 | 0.6940 | 0.4876 |
| Distribution breadth | The distance to county center | 0.8500 | -0.0249 | 0.0108 | 1.8970 | **0.0223** |
|  | Proportion of building area – 1000m | 0.5100 | -0.7139 | 0.4539 | 1.5720 | 0.1158 |
|  | Environmental noise | 0.2500 | -0.0097 | 0.0116 | 0.8370 | 0.4028 |
|  | Disturbance index | 0.1300 | 0.0925 | 0.1255 | 0.7370 | 0.4611 |

Appendix Table S10 The results of the chi-square test for differences in the number of species across three habitats (i.e., urban, suburban, and rural), categorized by distinct diets and nest sites.

|  | variables | χ² | df | p |
| --- | --- | --- | --- | --- |
| Diets | Omnivorous | 6.715 | 2 | **0.035** |
|  | Carnivorous | 5.481 | 2 | 0.065 |
|  | Insectivorous | 3.290 | 2 | 0.193 |
|  | Carnivorous & Insectivorous | 4.093 | 2 | 0.129 |
|  |  |  |  |  |
| Nest sites | Ground | 2.854 | 2 | 0.240 |
|  | Water | 1.660 | 2 | 0.436 |
|  | Crown | 0.000 | 2 | 1.000 |
|  | Shrubbery | 9.010 | 2 | **0.011** |
|  | Rock-Wall | 8.235 | 2 | **0.016** |
